# A method for generating an illusion of backwards time travel using immersive virtual reality—an exploratory study

**DOI:** 10.3389/fpsyg.2014.00943

**Published:** 2014-09-02

**Authors:** Doron Friedman, Rodrigo Pizarro, Keren Or-Berkers, Solène Neyret, Xueni Pan, Mel Slater

**Affiliations:** ^1^Sammy Offer School of Communication, The Interdisciplinary Center IDC HerzliyaHerzliya, Israel; ^2^Event Lab for Neuroscience and Technology, Departament de Personalitat, Facultat de Psicologia, Avaluació i Tractaments Psicològics, Universitat de BarcelonaBarcelona, Spain; ^3^Department of Computer Science, University College LondonLondon, UK; ^4^Institució Catalana de Recerca i Estudis AvançatsBarcelona, Spain

**Keywords:** time travel, virtual reality, body ownership, trolley problem

## Abstract

We introduce a new method, based on immersive virtual reality (IVR), to give people the illusion of having traveled backwards through time to relive a sequence of events in which they can intervene and change history. The participant had played an important part in events with a tragic outcome—deaths of strangers—by having to choose between saving 5 people or 1. We consider whether the ability to go back through time, and intervene, to possibly avoid all deaths, has an impact on how the participant views such moral dilemmas, and also whether this experience leads to a re-evaluation of past unfortunate events in their own lives. We carried out an exploratory study where in the “Time Travel” condition 16 participants relived these events three times, seeing incarnations of their past selves carrying out the actions that they had previously carried out. In a “Repetition” condition another 16 participants replayed the same situation three times, without any notion of time travel. Our results suggest that those in the Time Travel condition did achieve an illusion of “time travel” provided that they also experienced an illusion of presence in the virtual environment, body ownership, and agency over the virtual body that substituted their own. Time travel produced an increase in guilt feelings about the events that had occurred, and an increase in support of utilitarian behavior as the solution to the moral dilemma. Time travel also produced an increase in implicit morality as judged by an implicit association test. The time travel illusion was associated with a reduction of regret associated with bad decisions in their own lives. The results show that when participants have a third action that they can take to solve the moral dilemma (that does not immediately involve choosing between the 1 and the 5) then they tend to take this option, even though it is useless in solving the dilemma, and actually results in the deaths of a greater number.

## Introduction

We introduce methodology based on immersive virtual reality (IVR) that aims to provide an experience of travel backwards in time to relive a past event, and potentially change history. We further consider how such an experience might change attitudes and views of the virtual time traveler, given an illusory experience of going to the past and undoing actions that originally led to an unfortunate outcome.

In one view of today's physics time travel at the macro level to the past is not feasible, and even if it were apparently achieved it would be travel to a parallel universe (see extensive discussions in Deutsch and Lockwood, 2009; Deutsch, 2011). Nevertheless, the idea of time travel has long been one that has captured the imagination—both travel to the future in the story of *Urashima Tarô* (8C) and H.G. Wells' *The Time Machine*, and travel to the past (e.g., *Lest Darkness Fall* by L. Sprague de Camp). Time travel is a recurring theme in cinema; a recent example that included time travel is *Looper*^1^, involving the curious practice of criminals in the future sending their enemies to the past in order to be executed by paid assassins there.

Travel to the past raises scientific and philosophical issues that future travel avoids: it includes the possibility of changing of history with the paradoxes that this can imply. For example, in the Grandfather paradox, a person travels to the past and kills one of their own ancestors ensuring that they would have never been born to travel back to the past and accomplish this act. The movie *Looper* illustrates one of the potential paradoxes involved in time travel, where time travelers from the future can change history by amending the past, so that their own existence vanishes—leaving the viewer to wonder how any of the events of the movie could have taken place at all. Moreover, there have been recent attempts to discover whether time travelers are walking amongst us by examining whether the Internet contains any trace of their messages (Nemiroff and Wilson, 2013). Some of the paradoxes and philosophical arguments around the concept of time travel to the past are discussed, for example, in Grey (1999); Dowe (2000).

Irrespective of whether time travel to the past is in any way possible, here we consider the question—what if it were possible? What if someone could travel back through time and experience a sequence of events, and be able to intervene in order to change history? Specifically, we simulate a sequence of events that has a tragic outcome (deaths of strangers) in which the participant unavoidably plays an important role. In our setup the participant is caught in a classic moral dilemma: if he or she does nothing then five people would die for certain; if he or she acts then five people might be saved but another would die. This is derived from the moral dilemma known as the “trolley” or “boxcar” problem where an out of control trolley or boxcar on a railway track would kill five people unless diverted, in which case it would kill one. Such problems are usually addressed empirically by questionnaires^2^, where respondents are required to indicate whether they would do nothing allowing the five to die or divert the trolley thus killing one. Typically 80–85% of people choose to divert the trolley sacrificing one to save five (Hauser et al., 2007). Such classic moral dilemmas as the trolley problem have been studied in moral philosophy and more recently by neuroscientists in the context of the processes involved in action in moral decision making—see Greene et al. (2001); Cushman et al. (2006). Here we have simulated a moral dilemma similar to the trolley, except that the agent of harm is a (virtual) human rather than a run-away trolley, and where it is possible to find a solution to the problem without the deaths of anyone. Our work is exploratory considering whether the ability to go back through time and intervene, to possibly avoid all deaths through finding the solution, might have an impact on how the participant responds to such moral dilemmas, and also have an impact on how past actions with unfortunate outcomes in their own lives are seen.

We use IVR to give people the illusion of having traveled back through time. IVR can create three specific types of illusion that we exploited for this purpose: presence, body ownership and agency. The first, “presence,” is the illusion of being in the place depicted by the virtual environment (place illusion) and of the reality of the events taking place there (plausibility) (Sanchez-Vives and Slater, 2005; Slater, 2009). The second is the illusion of “body ownership”: in IVR it is possible to endow participants with an alternate life-sized virtual body that is spatially coincident with their own real body. This substitute virtual body is seen through a wide field-of-view stereo and head-tracked head-mounted display (HMD) from first person perspective when looking directly down toward the own body and also when looking in a mirror. This can give rise to the illusion that the substitute body is the participant's own body (Petkova and Ehrsson, 2008; Slater et al., 2010). Through real-time motion capture the virtual body moves synchronously with the real body, thus giving the third illusion (“agency”) (for example, as in Banakou et al., 2013) where the participant has the sensation of being the cause of the movements of the body. We aimed to create strong illusions of presence, body ownership, and agency, and using these to project a participant back through virtual time to relive and be able to intervene in a sequence of events over and over again.

If you are involved in a sequence of events and then step into a machine that takes you back to the start of those events, there are various possibilities regarding what you would perceive and how you might be able to act. Of course you should see the events that had occurred, which includes perceiving that earlier version of yourself doing whatever you originally did, together with all the consequences of those actions. However, immediately there is a problem: can you intervene in and change those events or not? If you have physical presence then you can cause events just as any other agent in the situation. This physical presence also implies that your former self should be fully aware of you. However, were this to be the case then the first time that you had experienced those events your future self should have been present, and you should have a memory of that having occurred. Alternately you may have no physical presence but just be an observer of those past events. This type of situation is discussed in Deutsch (2011) where this example is used to show that the possibility of physical intervention requires the existence of parallel universes (the Multiverse). Here, since it is virtual reality, and we are not bound by the laws of physics governing time travel, we can adopt an intermediate position. You as the time traveler can effect changes to the events, therefore changing history, but you are not visible to your past self.

We use two different conditions in our exploratory study. In both participants are embodied in a virtual body and experience a sequence of events in which they are faced with the choice of allowing five people to die or saving the 5 at the cost of 1. In the *Time Travel* condition at the end of this sequence they are transported back to the start of the events, but see and hear their previous self-representation carrying out the actions that they had carried out before. In this condition they can cause events, but the previous incarnation of the self is not aware of the current self. Subsequently once the sequence of events is played out again, possibly changed from the first time around due to new events caused by the participant, the participant is once again transported back to the start, now seeing and hearing the two previous self-incarnations. Thus, there are three trials. In the *Repetition* condition the first trial is the same as the Time Travel condition, but in the second trial the participant is simply faced with exactly the same events again, as in a video game where a “life” is lost and the game starts again. Here there is no notion of “time travel”—the representations of the previous incarnations are not shown, and the participant is free to act, in the second and third trials of course utilizing the knowledge gained from the previous trials. It should be noted that the first trials in both conditions are therefore identical and provide an IVR experience representing a version of the classic trolley-type of moral dilemma.

The goal of this exploratory study was to discover (i) whether the Time Travel condition would be more likely to result in the illusion of time travel than the Repetition Condition, (ii) the extent to which the experience of illusory time travel might influence attitudes toward morality, moral dilemmas and “bad decisions” in personal history.

The thinking behind our approach is that at some level the brain does not distinguish between reality and virtual reality. Therefore, there would be implicit learning that the past is mutable. The illusion that the past can be changed might have important consequences for present day attitudes and beliefs including implications for psychotherapy.

## Methods

### The scenario

**Figure 1:**
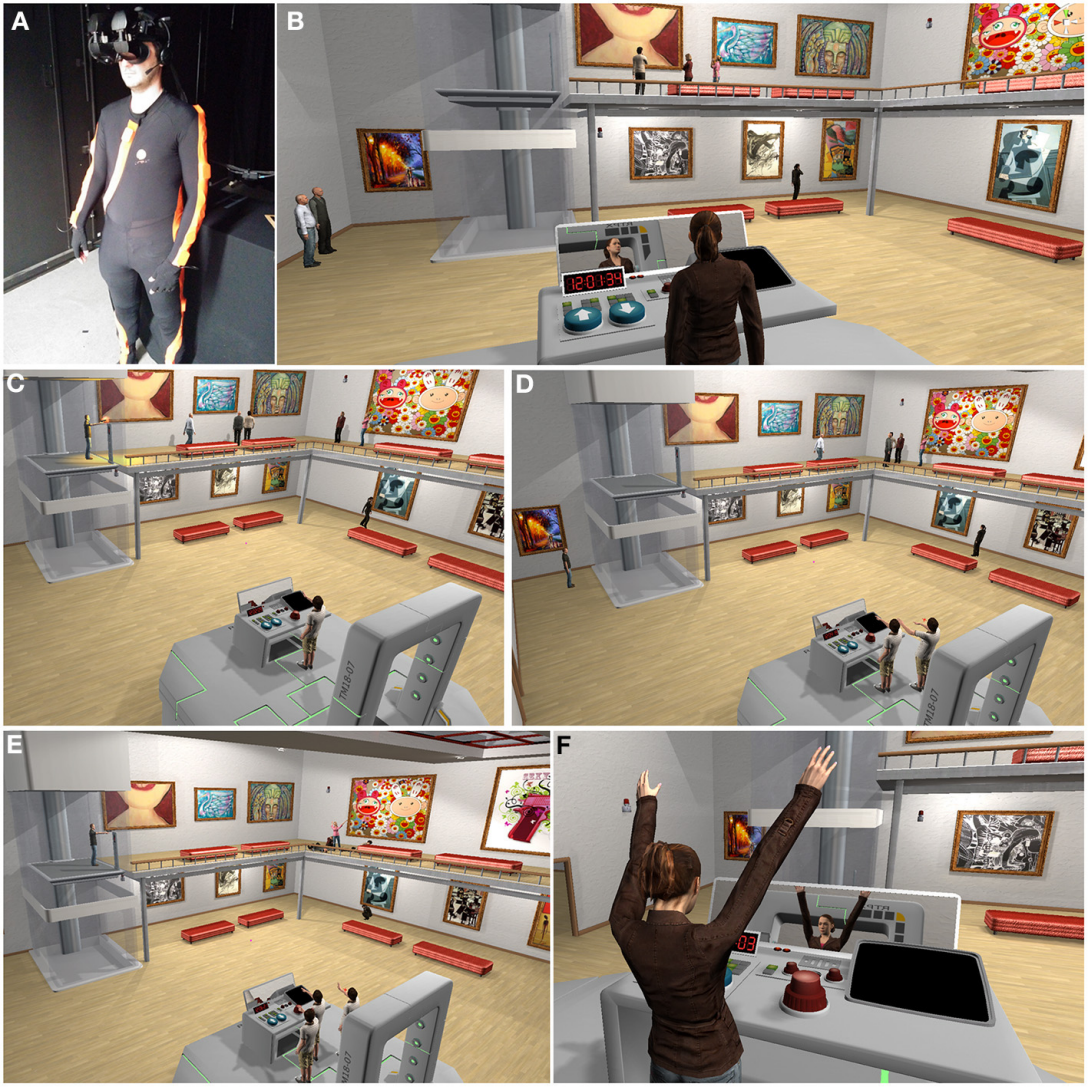
**The gallery room scenario. (A)** A participant wearing the head-mounted display and the motion capture suit. **(B)** The gallery with three visitors at the upper level, 1 on the ground level, and 2 waiting to be taken up. The workbench is shown with the up and down elevator control buttons and the red alarm button. The mirror reflects the virtual body of the participant (here female), which can also be seen from behind. The participants saw the environment from the first person perspective of the body, and the virtual body was coincident in space with their real body. **(C)** The gunman shoots at the five people on the upper level. **(D)** The time travel (2nd time around)—where the participant is embodied in the rightmost body by the workbench, and sees his previous self-carrying out the actions from the 1st time around. **(E)** The time travel 3rd time around, where the gunman is shooting. The participant is in the leftmost body behind the workbench, and the two earlier clones are to his right. **(F)** A close up of the embodiment illustrating visuomotor synchrony. Here the participant sees through the eyes of the virtual body and as she raises her arms the arms of the virtual body raise synchronously, and this is also seen in the mirror reflection.

The specific scenario we created was an art gallery on two levels (ground and upper) (Figure 1). This is based on earlier work on action in response to a moral dilemma (Pan and Slater, 2011). In this situation the participant learns to operate a virtual elevator that takes (virtual human) visitors to the upper level or down from upper level to the ground level at their request, and also learns to operate an alarm that freezes the elevator in place and makes an alarm sound. After six visitors have entered the gallery there are five people browsing the paintings upstairs and one person downstairs. A seventh person enters the gallery and asks to be taken to the upper level. Upon arrival at the upper level, and while still on the elevator platform, he immediately takes a gun out of his pocket and starts shooting at the five people there. The participant (elevator operator) then has a choice to make: either leave the gunman to possibly kill all five people or send the elevator down, where the one person might be killed instead. The participant has also previously learned that pressing an alarm button will immediately freeze the elevator in place (but this is no use at this moment since the gunman is already shooting). After a few seconds of this mayhem the scene dissolves and the participant is back at the start of the whole sequence of events. Note that one potential solution to the dilemma is to trap the gunman on the elevator half way between the two floors by pressing the alarm button. However, the first time that participants experience this sequence of events this possibility is not useful, since they do not know that the seventh person is a gunman until he is already shooting. Therefore, if the goal were to save the five visitors on the upper level then freezing the elevator in place would be exactly the wrong thing to do since it would leave the gunman in the position to shoot all of them.

In the Repetition condition exactly the same sequence of events will then reoccur. In this condition the participant can choose how to act now with knowledge about what is likely to happen, and can of course change his or her actions compared to the first “life.” In the Time Travel condition, however, each participant sees and hears the earlier incarnation of him or herself performing what they had actually done in the first round. We refer to this virtual human character, that has the identical virtual body as the participant, and that re-enacts the first round actions of the participant as P1, and the actual human participant as P. P1 does the same actions as the participant had actually done and is unaware of P. If P does not take any action (i.e., pressing buttons) the sequence of events will unfold the same way as in the first round, otherwise any action can result in a different sequence of events and outcome.

Eventually in this second round there may be five people on the upper level and one on the ground level (depending on the actions of the participant) as before, and the gunman enters again. The participant might infer that the same is likely to happen as in the previous round and therefore try to take steps to stop this. In particular, P might realize the solution, and try to trap the gunman between levels. In any event, whatever happens, once again after the shooting takes place (or if the gunman is trapped) the scene dissolves and the participant is back at the start. For those in the Repetition group this is the start of the third identical trial. However, those in the Time Travel condition now see their two previous selves (P1 and P2) doing what they had done before. Each participant finally experiences the three trials, and then the virtual reality part of the experiment is terminated.

### The abstract representation of time travel

Our approach to time travel is designed to provide participants with the illusion of time travel. We will show how our approach is different from typical simulations or video games, in which the player can repeat a game multiple times. In this section we explain our approach and illustrate it in relation to the gallery scenario described above.

There are two levels of abstraction: the logic layer and the VR layer. The logic layer is an abstraction handled by an automated reasoning engine, which deals with the unfolding of the narrative, and the VR layer has a much richer description of the virtual world, which supports the immersive experience of virtual time travel.

In the art gallery scenario the virtual environment includes the following objects: visitors in the gallery, the participant, the elevator, a workbench with a mirror, up and down buttons to control the elevator and an alarm button to freeze the elevator. The participant is instructed to operate the elevator and follow the visitors' requests as described earlier, and at the end of the scenario, after the shooting, is transported back in time (or to replay the scenario again in the Repetition condition). Here we concentrate on the Time Travel condition since the repetition condition only involves running the same program three times. We refer to the original scenario as the 1st time around, and after the time travel to the past, the 2nd time around, and then the 3rd time around. The destination time was always fixed—time travel back to the beginning of the scenario. However, the system does support time travel to arbitrary times in the past.

Following time travel to the past the participant is embodied in a new virtual body (looking identical to the first one). We will refer to the virtual bodies representing the participant in the past as clones. The participant sees and hears him- or herself from behind and to the right side of the previous clone (Figure 1D, Supplementary Movie S1). Everything takes place exactly as it did in the 1st time around, until the participant takes actions that modify the story line. Our system allows multiple time travels and thus supports a large number of co-existing clones, and in this case two past selves during the 3rd time around.

The crux of the time travel approach is the reasoning engine, which is able to track and maintain causality. Causality raises many scientific and philosophical questions. In this paper we adopt the common sense notion of causality, which has also been the subject matter of empirical studies (e.g., Michotte, 1963). In our case the only way for the participant to “change the past” is by controlling the elevator (directly by pushing its buttons or indirectly by activating and deactivating the alarm). The reasoning engine can deduce, for example, that if the gunman is not on the upper floor then he cannot shoot the visitors in the upper floor, and that if the visitors are not killed then they remain alive.

Our system tracks causality by maintaining an abstract representation called *history*, which consists of a sequence of states and a sequence of actions. At each moment the virtual world is in some well-defined state, and objects perform actions that transfer the world from one state to another. The reasoning engine attempts to keep the history of the 2nd time around as close as possible to the 1st time around, but it is also takes into account the new actions introduced by the participant in the 2nd time around, together with their causal effects.

All objects are defined by their state. For example, a visitor is defined by its location and whether it is alive or dead, and the elevator's state describes whether it is in the ground floor, upper floor, or traveling in between floors. An important challenge is to include in the reasoning engine only those state variables and actions that are necessary for the reasoning process. There are many details that need to be modeled in the VR but for tractability we can assume they do not affect the narrative, and thus are not included in the reasoning engine. For example, in the abstract specification it is not important exactly where the visitors are, so in terms of the reasoning engine their location is abstracted to being either outside the gallery, on the ground level, or on the upper level. In the VR layer exact trajectories in space and time are maintained.

We distinguish between three types of entities, in terms of causality: (i) the participant, (ii) physical objects, and (iii) agents. The latter category includes, in our case, both the visitors and the previous clones of the participant. The human participant is always assumed to have “free will”; the system cannot dictate his actions, and cannot override his previous actions. Second, there are simulated objects; in our case, the elevator and the alarm. We assume that physical objects follow well-defined deterministic rules, and therefore our engine applies standard simulation techniques to these objects.

The main difference between our approach and other simulations is the way we model human behavior; this is the third class of entity—the agents. For these entities the algorithm tries to “replay” their behavior as much as possible, i.e., simulated humans will act during the 2nd time around exactly as they did the 1st time around, unless this involves a logical contradiction. This part of our method is again consistent with our common sense view; even if we have approximate models of human behavior, sometimes we regard people's actions as idiosyncratic and arbitrary. It is exactly this seemingly unpredictable behavior that makes narratives compelling, and this is what we want the time travel approach to maintain. In our case such time travel modeling is restricted to humans, but in general there can be other low probability events that we want to be repeated the 2nd time around in the same way and at the same moment that they happened in the 1st time around. In our case we have opted to model the previous clones of the participant as a type of agent, just like the visitors.

Technically, all actions have *preconditions* and *postconditions*. Preconditions define the states that specific objects should be in, so that the action can be performed. Post-conditions define the specific states that specific objects should be in after the action is performed. A major element of the time travel approach is that the 2nd time around the system tries to repeat the history exactly as it took place the 1st time around, but if some preconditions do not hold for specific actions then these actions do not take place. In some cases the reasoning engine replaces these actions by similar actions, and in other cases these actions are avoided altogether.

During the 1st time around the system plays events according to a predetermined script and records the participant's actions. The reasoning engine is only activated the 2nd time around, or, in general, after the first occurrence of time travel to the past. From that point onwards the simulation engine operates differently for three types of actions: (i) new actions taken by the participant, (ii) actions performed by simulated physical objects, and (iii) actions that were taken by agents and need to be repeated following what happened the 1st time around.

If the participant takes an action it is always executed—if the participant was able to take this action in the VR then it cannot have been logically invalid. The action is also recorded in the history of the 2nd time around; this is necessary for further time travels (in our case there is also 3rd time around). A second category consists of those actions taken by simulated physical objects. In our case this applies to the elevator and the alarm—they are simulated in the 2nd time around regardless of the 1st time around. If the participant does not intervene then the alarm and the elevator would behave exactly as they did in the 1st time around. Also, if we would have attempted to replay the elevator's actions in the same way that we model agents then any slight divergence of the 2nd time around from the 1st time around might result in incoherent behavior. This discrepancy in the way we model physical objects and human agents is in accordance with common sense and everyday psychology: as people we pay much attention to human behavior and its timing, but we do not pay as much attention to automated objects, as long as they behave consistently.

The third category of actions consists of those that were taken by agents the 1st time around and now need to be repeated. Following time travel the virtual time is reset to the beginning of the 1st time around. As time passes (both virtual and physical time), whenever the virtual time coincides with the virtual start time of a recorded action, the reasoning engine checks the preconditions of this action. If these are satisfied then the action takes place, exactly as it did the 1st time around. However, if any of the preconditions are violated, the action does not take place. Instead, the reasoning engine tries to automatically replace it by a similar action, as explained below. If no replacement is possible, the action is ignored.

In our scenario there are only two types of actions that can be replaced by other actions. The main automated replacement is for the shooting action by the gunman. The 1st time around the gunman shoots the five visitors in the upper floor, let us label them V_1_, V_2_, V_3_, V_4_, and V_5_. The 2nd time around the gunman tries to shoot the same number of visitors at the same (virtual) time. If the 2nd time around the gunman is again at the upper floor then the shooting takes place as it did in the 1st time around. However, if the gunman is now on the ground floor the reasoning engine tries to match new targets for the shooting actions. The result is that the gunman shoots the single visitor that is on the ground floor, and even the participant. Since there is only one visitor in the ground floor, V_6_, the reasoning engine replaces V_1_ by V_6_ and V_2_ by the participant. The other visitors, V_3_–V_5_, cannot be replaced, so these actions are omitted from the history.

A similar action correction takes place for the action “enter-floor,” which takes place anytime that any of the visitors (or the gunman) exits the elevator. If the engine reaches a time that the visitor has to exit the elevator, that visitor is in the elevator, and the elevator is stationary at one of the floors, then the visitor exits the elevator, regardless of whether it is the same floor as the 1st time around.

There are two “solutions” that the participant can reach in order to prevent any shooting. The first solution is to let the gunman enter the elevator but press the alarm button before the elevator reaches the upper floor. The second solution is to avoid sending the visitors upstairs, but to send the gunman upstairs. Quite a few participants were able to arrive at the first solution (see Results), but none realized the second solution.

An additional solution would be to keep the gunman on the ground floor. However, in this case the gunman would always shoot at least one visitor—the one that was preprogrammed to remain in the ground floor—and possibly the participant.

We provide this narrative as a concrete example illustrating the reasoning engine in action. Consider the following abstract script fragment, which takes place after the gunman enters the gallery, 1st time around (G is the gunman, P is the participant, V_1_,…,V_5_ are the upper floor visitors):


G enters gallery
G enters elevator
P pushes elevator up
Elevator starts moving up
Elevator arrives upper floor
G shoots V_1_, V_2_, V_3_, V_4_, V_5_
P pushes alarm
alarm on
P travels back in time


Second-time around the participant tries to avoid the shooting by turning on the alarm, just after his previous clone (now P_1_) sent the elevator up. The new action and state are shown in bold.


G enters gallery
G enters elevator
P1 pushes elevator up
**P pushes alarm**
**alarm on**
Elevator starts moving up
Elevator arrives upper floor
G shoots V_6_
P1 pushes alarm
*alarm off*


As a result the elevator is blocked and does not go to the upper floor; this is marked in the two actions shown as crossed out: they happened 1st time around but not 2nd time around. The reasoning engine then replaces the shooting of the visitors in the upper floor by another action—shooting the visitor on the ground floor—this replaced action is shown as underlined. Finally, note that the alarm object is simulated, so now after the shooting it is actually turned off by the clone P_1_ rather than being turned on as the 1st time around (shown in italic). This is because the alarm is a toggle (pressing it will change its state to the opposite state) and so since the participant had turned it on, when the clone presses the button again it will be turned off. Here actions are preserved rather than outcomes.

In this explanation we have concentrated on the 1st time and 2nd times around but our system allows multiple time travels. Whenever history has changed by time travel it now becomes the relevant history for the next time travel. That is, whenever we write 1st time around and 2nd time around these could be replaced by (*n*-1)th time around and *n*th time around, respectively. Or in other words: whenever history “changes” the original history is discarded and the new history becomes the frame of reference.

An important part of our method is that the reasoning engine is integrated with the IVR system. Where the reasoning engine decides, for example, “the gunman shoots at visitor 1” this has to be transformed into actual animations of the virtual human characters, who are in a particular place in a relatively complex scenario, with a certain body size, a certain distance between them, and so on. Describing how the VR layer is combined with the reasoning engine is beyond the scope of this paper, and will be discussed in later work.

## The experiment

### Participant recruitment

Thirty-eight participants were recruited for the experiment. Each was assigned on arrival and alternately to one of the two groups (Time Travel or Repetition) by order of their appearance at our laboratory. Each participant was asked to attend the laboratory on two occasions separated by approximately a week. The sample had an equal number of males and females in each of the two groups. However, the results of six participants were discarded (four due to technical problems and two because they failed to attend the second session of the experiment). Therefore, the final sample size was 32, 16 in each group, with an even gender balance. There were no differences between the groups with respect to mean age, prior use of virtual reality, gaming experience, etc. (Supplementary Material 1).

The study was approved by the Bioethics Committee of the University of Barcelona. All participants were given basic information about the experiment (the real purpose of the study was not revealed prior to completion) and signed an informed consent form when they agreed to take part of each phase of the study. They were paid 5€ for participating in the first session of the experiment, and 5€ more when they came back for the second part.

### Equipment

The virtual environment was implemented in Unity3D and delivered visually through a wide field-of-view stereo HMD the NVIS nVisor SX111^3^ (Figure 1A). This has dual SXGA displays with 76°H × 64°V degrees field of view (FOV) per eye, totaling a wide field-of-view of 102° horizontal and 64° vertical, with a resolution of 1280 × 1024 per eye displayed at 60 Hz. Head tracking was performed by a 6-DOF Intersense IS-900 device. Participants wore Asus HS-1000W earphones^4^ over the HMD.

Tactile feedback was provided based on an Arduino board connected to the computer via USB controlling two small vibrator devices. The vibrator devices were placed in the palmar areas of the middle fingers of each hand to give vibrotactile feedback when the participant touched the buttons that control the elevator in the virtual environment.

Participants in the art gallery were endowed with a gender-matched human virtual body. This moved in real time with the movements of the participant. In order to achieve this we used an Xsens body tracking suit for motion capture and MVN Studio software. Hence movements of the virtual body were mapped in real-time from the motion capture of the participants' real movements.

Based on results from previous papers (Banakou et al., 2013; Peck et al., 2013; Kokkinara and Slater, 2014) we expected high scores on a questionnaire that assessed the illusions of body ownership (the virtual body perceptually experienced as the own body) and agency (the sensation of causing the movements of the virtual body). In this experimental setup participants would have both visual-motor synchrony (through the motion capture) and some visual-tactile synchrony (through the vibrotactile stimulation on the palms of the hands whenever they touched the buttons to control the elevator).

### Procedures

On their first visit to the laboratory participants signed an informed consent form, and completed a demographic questionnaire (giving information about their age, work, and so on) and two implicit association tests (see Section “Implicit Association Test for Morality” and Supplementary Material 4). They then entered the virtual environment using the head-tracked HMD and earphones. They heard pre-recorded instructions through the earphones to look around and describe the environment. They first saw a simple training environment, and then the art gallery, with some virtual visitors arriving and going to the upper level or staying on the ground floor looking at the paintings. In this experience they were embodied in a virtual body as described earlier, but did not wear the motion capture equipment, so they were asked to sit in a specific posture, corresponding to that of their virtual body. After exiting the virtual environment they were asked to secretly rate three past decisions in their lives that they regretted and the information was placed in a sealed envelope unseen by the experimenters. They were then asked to read a short passage about the meaning of time travel. The aim of this was to instill the idea that when past history is changed it means that the original history actually never happened (Supplementary Material 5). Finally the participants were paid for their attendance at the first session. There were two experimenters present throughout. Procedures are illustrated in the Supplementary Movie (this is in three parts only for reasons of space).

Approximately 1 week later they returned to the laboratory, and during this time they experienced the full scenario described in Section “The Scenario,” according to the condition to which they had been assigned. Prior to the start of the scenario they first learned how to control the elevator to take visitors to the upper floor, and to bring the elevator down again. They controlled the elevator by pressing up and down buttons on a virtual workbench in front of them. They also learned about the red alarm button on the workbench, and that it would freeze the elevator and emit an alarm sound. They learned that the button was a toggle that would switch on the alarm, or switch it off if the alarm was on.

In this second week's exposure they were standing with full body tracking, saw their virtual body by looking at it directly from first person perspective, and also in a mirror that was part of the workbench. Since they were wearing the motion capture suit there was visual-motor synchrony between their movements and that of their virtual body. When they touched the up, down or alarm buttons they would see their virtual body move accordingly and feel a corresponding vibrotactile sensation on the palm of the active hand.

It is important to realize that in subsequent replays the participants not only saw their previous incarnations carrying out their former actions but also heard themselves speak as they spoke before. For example, the 1st time around one of the visitors always asked the participant for the time. Thus, the subsequent times around the participants could hear their own voice replayed.

A particular trial in both the Time Travel and Repetition conditions was terminated either 7 s after the last shooting and when the gunman did not have a possibility of shooting again (i.e., the elevator was not moving and there were no visitors alive on that level), or when the gunman had been trapped inside the elevator for 7 s. Then the operator would trigger the next trial, or at the end of the third trial terminate the experiment.

At the end of their three experiences the participants took off the HMD and they completed the same two IATs as in their first visit. They then completed a questionnaire concerned with the illusions of body ownership, agency, presence, the illusion of time travel, and other aspects of their experience. They were then asked to think about their three bad decision ratings that they had made the previous week, asked to rate these decisions again, with the sealed envelope available to them. The experimenter was able to record the score from the previous week and the new score, though at no time knew what the decisions were about. After this the participants were interviewed and debriefed, removed all the equipment, and were paid.

The full procedures are described in detail in Supplementary Material 2.

### Response variables

#### Body ownership, agency, and presence

Since this is an exploratory study intended to introduce how IVR can be used to generate an illusion of time travel, here we only report the variables that ultimately proved useful in our exploratory statistical model of the results. Details of the variables measured are given in Supplementary Material 3.

The perceptual illusion of ownership with respect to the virtual body seen from first person perspective that substituted the real body was assessed with four questions in the questionnaire administered after the three scenario trials. Each question was in the form of a statement that was rated on a 1–7 Likert scale where 1 represented “strongly disagree,” and 7 “strongly agree.” The questions are shown in the Body ownership section of Table 1. These questions were based on previously published work (e.g., Banakou et al., 2013).

**Table 1:** **Questions for body ownership, agency, presence, and guilt**.

**Concept**	**Variable name**	**Statement**
Body ownership	*mirror*	Even though the virtual body I saw did not look like me, I had the sensation that the virtual body I saw in the mirror was mine.
	*down*	Even though the virtual body I saw did not look like me, I had the sensation that the virtual body that I saw when I looked down at myself, was mine.
	*other*	I felt that the virtual body that I saw was someone else.
	*mybody*	Overall even though the virtual body I saw did not look like me I had the sensation that the virtual body I saw was my body.
Agency	*agency*	The virtual body moved according to my movements.
Presence	*placeillusion*	I had the sensation of being in the gallery
	*plausibility*	There were times when the gallery was more real for me than the laboratory in which everything was really taking place.
	*copresence*	How much did you find yourself responding to the visitors as if they were real people?
Guilt and self-assessment	*guilt*	Do you feel any guilt about what happened to the visitors?
	*triedmybest*	I tried my best to save the visitors from the shooting.

*All responses on a 1–7 scale with 1 meaning most disagreement with the statement, and 7 most agreement*.

In order to obtain one overall score a factor analysis was carried out on the four variables (*mirror, down, other, mybody*) using principle components factors (Stata 13^5^). This resulted in one factor with the best fit to the original data with the smallest uniqueness values per variable, and explaining 76% of the total variance. Table 2 shows the factor loadings and uniqueness values. From the factor analysis a combined score (*Ownership*) was derived using the regression scoring method. The factor loadings in Table 2 are equivalent to the Pearson correlations between *Ownership* and the original four variables.

**Table 2:** **Factor analysis for body ownership questions**.

**Variable**	**Factor loading**	**Uniqueness**
*mirror*	0.94	0.11
*down*	0.78	0.40
*other*	−0.79	0.38
*mybody*	0.96	0.08

Agency was assessed with the *agency* question. The main point of this was to test the adequacy of the real-time motion capture and display of the virtual body. Whereas due to individual differences there could be variations amongst participants in relation to body ownership, we did not expect much variation with respect to agency since it was a factual statement that the body did (or did not) move according to the movements of the participants. In fact 28 out of the 32 participants rated the agency question with a score of 6 or 7, and the remaining 4 with a rating of 5. We will therefore not refer again to this variable, it is simply an indication of system performance.

The illusion of presence (“place illusion” and “plausibility”) was assessed with the questions shown in Table 1. Again these were taken from previous papers—see Box 2 of Sanchez-Vives and Slater (2005). The “copresence” question was included to assess how participants assessed the virtual visitors. The *copresence* scores are uncorrelated with both *placeillusion* and *plausibility*, but Spearman's rho = 0.39 (*P* = 0.03) between *placeillusion* and *plausibility*.

Table 1 also includes questions about “guilt” and whether participants felt that they “tried their best,” that proved useful in the analysis. These were not taken from any existing source.

#### Implicit association test for morality

There were two IAT results from each of the two visits to the laboratory. Both are described in detail in Supplementary Material 4. One was concerned with feelings of guilt based on Xu et al. (2012). However, the Guilt IAT taken after the second visit VR experience resulted in 5 missing values due to procedural or participant errors and so could not be used. The second was based on Perugini and Leone (2009) concerned with the moral behavior of the participant. This IAT has been shown to correlate well with actual moral behavior. There were no missing values in these data. We refer to the two moral IAT scores as *PreIAT* and *PostIAT* for the scores in the first and second week respectively (the second of course taken after the VR experience).

#### Discomfort about 3 past decisions

The three bad decisions rated by the participants in both the first week and after the end of their experience in the second week were each rated on a 1–100 scale, representing their degree of regret about those decisions (100 the greatest regret). We calculated the mean of the three decision scores each week. The corresponding variables are *PreRegret* for the mean score in the first week, and *PostRegret* for the mean score at the end of the VR experience in the second week.

#### Moral choice scenarios

At the end of the questionnaire the participants read five moral dilemma scenarios—three were based on the boxcar problem (equivalent to the trolley problem), and two were based on the actual dilemma in the gallery (Supplementary Material 3). For each one they were asked whether or not they would “push the switch”—in each case resulting in the deaths of 5 people or 1. We restrict attention to the boxcar problem of which there were three variants each with a yes/no answer.

- Boxcar 5—the boxcar by default will kill 5, throwing the switch will divert the boxcar to kill 1 instead. Question: would you throw the switch?
- Boxcar 1—the boxcar by default will kill 1, throwing the switch will divert the boxcar to kill 5 instead. Question: Would you throw the switch?
- Boxcar footbridge—the boxcar by default will kill 5. If a man with a heavy backpack is pushed onto the track from a footbridge where he and the observer are standing then the 5 will be saved but the man will be killed. Question: would you push the man off the footbridge onto the track?

From these three we construct a new variable representing the number of scenarios out of the three in which 1 will be saved rather than the 5 [pushing the switch in (a), not pushing it in (b) and not pushing the man in (c)]. We refer to this variable as *save1*, which ranges from 0 to 3. Also we single out the footbridge question since this has a different element involving actively killing 1 by pushing him to save 5. Hence we will also use *footbridge*, which is a binary variable, in place of *save1*.

#### The illusion of time travel

This was assessed with the question: “The overall experience was more like…” where the response was on a 1 to 7 scale, 1 meaning “Replaying a video game” and 7 meaning “Experiencing time travel.” We refer to the corresponding ordinal variable as *timetravel*.

#### Hypotheses and statistical methods

This exploratory study was motivated by the idea that a strong sense of presence and body ownership in a virtual environment, together with a scenario where participants witnessed their own past actions carried out by a virtual human in which they had previously been embodied, would lead to an illusion of having traveled in time. A second level hypothesis was that the illusion of traveling in time might influence present day attitudes—in particular possibly lessening negative feelings associated with past decisions, giving a different perspective on past actions—including those associated with the experienced scenario.

This being a new area of study we collected a large amount of data (Supplementary Material 3). We focus here only on the core ideas.

For analysis we used statistical models appropriate to the type of data. *Condition* is a binary factor (Repetition = 0, Time Travel = 1), similarly with Gender (Male = 0, Female = 1). We treat *Ownership* as a continuous latent variable representing the subjective illusion of ownership of the virtual body, and positively associated with *mirror, down, mybody*, and negatively associated with *other* (Tables 1, 2).

*PostIAT* and *PreIAT* are treated as continuous variables, where greater values are associated with more moral behavior (Supplementary Material 4). *PreRegret* and *PostRegret* are likewise treated as continuous variables with greater values indicating greater discomfort about the three past decisions. Our model fitting strategy allows the “Post” variables to be influenced by the “Pre” variables, in other words the “Pre” variables appear on the right hand sides of the equations defining the model.

*Save1* is a variable representing a count out of a maximum of 3. We treat this as a binomial random variable, where greater values indicate a greater propensity to save the 1 (non-utilitarian) rather than the 5 (utilitarian). *Footbridge* is treated as a binary (Bernoulli) random variable, 1 indicating a “yes” answer to pushing the man to stop the boxcar (utilitarian), and 0 “no” (non-utilitarian).

The remaining variables are all questionnaire responses measured on an ordinal scale from 1 to 7.

Stata 13 was used for all statistical analysis. Since we have a multilevel hypothesis (condition, presence and body ownership influence time travel, and time travel in turn influences various other responses) we use path analysis to bring all relationships of interest into one statistical model. Stata 13 has the facility for path models that include factor variables and handles distributions other than normal (the function “gsem”). Path analysis was used since it supports the simultaneous evaluation of multiple stochastic equations—in other words it is not restricted to a single response variable as in the case of the general linear model (regression, ANOVA). Given the specification of any model (i.e., set of stochastic equations) the total covariance matrix is estimated, typically through maximum likelihood estimation. Path analysis was first described in the 1920's (Wright, 1921) with an up to date explanation in, for example, (Kaplan, 2009). We have used this several times before to unravel complex relationships in the context of body ownership studies (e.g., Kilteni et al., 2012; Llobera et al., 2013; Pomes and Slater, 2013; Steptoe et al., 2013). Given the nature of our study, path analysis has been used as an exploratory rather than a confirmatory tool.

The path model specifies each variable according to its type as discussed above. In particular the equations specifying continuous variables are assumed to be normal linear models, the binary, and count variables are treated as binomial-logistic models, and the ordinal questionnaire data as ordered logistic models. Throughout we have used robust standard errors of the coefficient estimates since these allow departure from the strict distributional assumptions underlying the statistical inference models. Moreover, we also relax the assumption of independence between observations, allowing for the fact that there may be less variation in responses within each gender group than between them—in other words we use robust standard errors allowing for clustering on gender.

## Results

### Presence and body ownership

**Figure 2:**
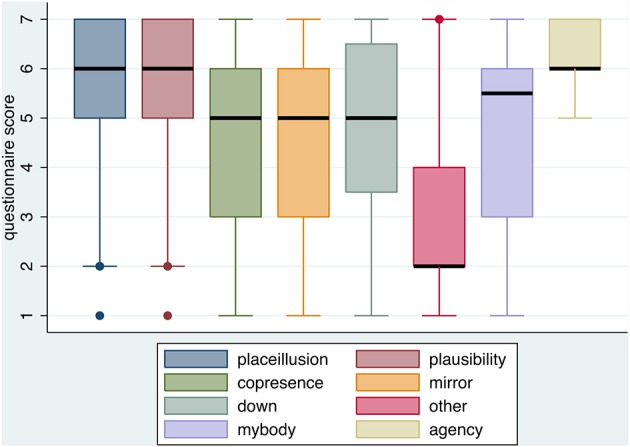
**Box plots for the presence and body ownership and agency illusion questions**. The thick horizontal lines are the medians, and the boxes the interquartile ranges. The whiskers extend to 1.5 the interquartile range or the extreme values in both directions. Values outside of this are shown as single point outliers.

Figure 2 shows box plots for the presence, body ownership and agency questions (Table 1). It can be seen that the subjective levels of presence were high (median of 6 and interquartile range 5–7 for both *placeillusion* and *plausibility*) and slightly lower (median 5) and greater variability (IQR 3 to 6) for *copresence*. The body ownership illusion scores were relatively high (median 5 for each of the positive questions, and 2 for the control question *other*) well in line with previous studies (e.g., Banakou et al., 2013). The *agency* scores were high as discussed earlier.

Overall the goal of producing a system that could lead most participants to a high level of presence, body ownership and agency, was achieved, though of course with variation due to individual differences. There were no overall differences on any of these variables due to condition (Repetition, Time Travel) or gender.

### How participants addressed the moral dilemma

As noted earlier in the first round all participants faced the moral dilemma of doing nothing, in which case 5 would die, or sending the elevator down potentially to endanger 1. There were possible solutions that would have avoided all deaths, but this could not have been known to any of the participants the first time around since they did not know at this stage that the seventh visitor was a gunman until he started shooting.

The one difference with the classical moral dilemma (such as the trolley or boxcar) was that there was a third, albeit useless, action that participants could take once the shooting started the 1st time around—press the alarm. In fact 28 out of 30 subjects selected this as their first action, and 2 selected the Down button (to save the 5) (data on 2 participants was not available). For their second action 12/30 (40%) of participants chose to press the Down button. For their third action 6/30 pressed the Down button. By the end of the sequence in only 1 case was the elevator down though, due to attempts by participants to trap the gunman and thus moving the elevator up and down, and the remaining times it was up (64%) or between floors (32%).

The 1st round resulted in 24/30 (80%) of cases where 5 visitors were shot. The mean and standard deviation of the number shot is 4.8 ± 0.81.

During the second round participants carried out almost double the number of actions in the Time Travel condition compared to Repetition (Table 3), and the difference is significant (Wilcoxon rank-sum test, *P* = 0.012). This difference is due to the Time Travel participants having to cope with the 1st round actions taken by their earlier self. In this condition the numbers shot were almost the same between the two conditions (Table 4).

**Table 3:** **Mean and standard errors of numbers of actions by condition**.

**Condition**	**2nd time around**		**3rd time around**	
	**Mean**	***S.E*.**	**Mean**	***S.E*.**
**Repetition**	2.1	0.51	2.1	0.52
**Time travel**	4.5	0.74	3.5	0.58

**Table 4:** **Mean and standard errors of numbers shot by condition**.

**Condition**	**2nd time around**		**3rd time around**	
	**Mean**	***S.E*.**	**Mean**	***S.E*.**
**Repetition**	2.3	0.48	1.2	0.43
**Time travel**	2.2	0.58	1.5	0.49

The third time around the number of actions in the Time Travel condition reduced to be not much more than the Repetition. This is because participants who had found a solution the second time round could just let this play out again. The numbers shot also decreased again (Table 4).

We consider these results in the Discussion.

### Path analysis

**Figure 3:**
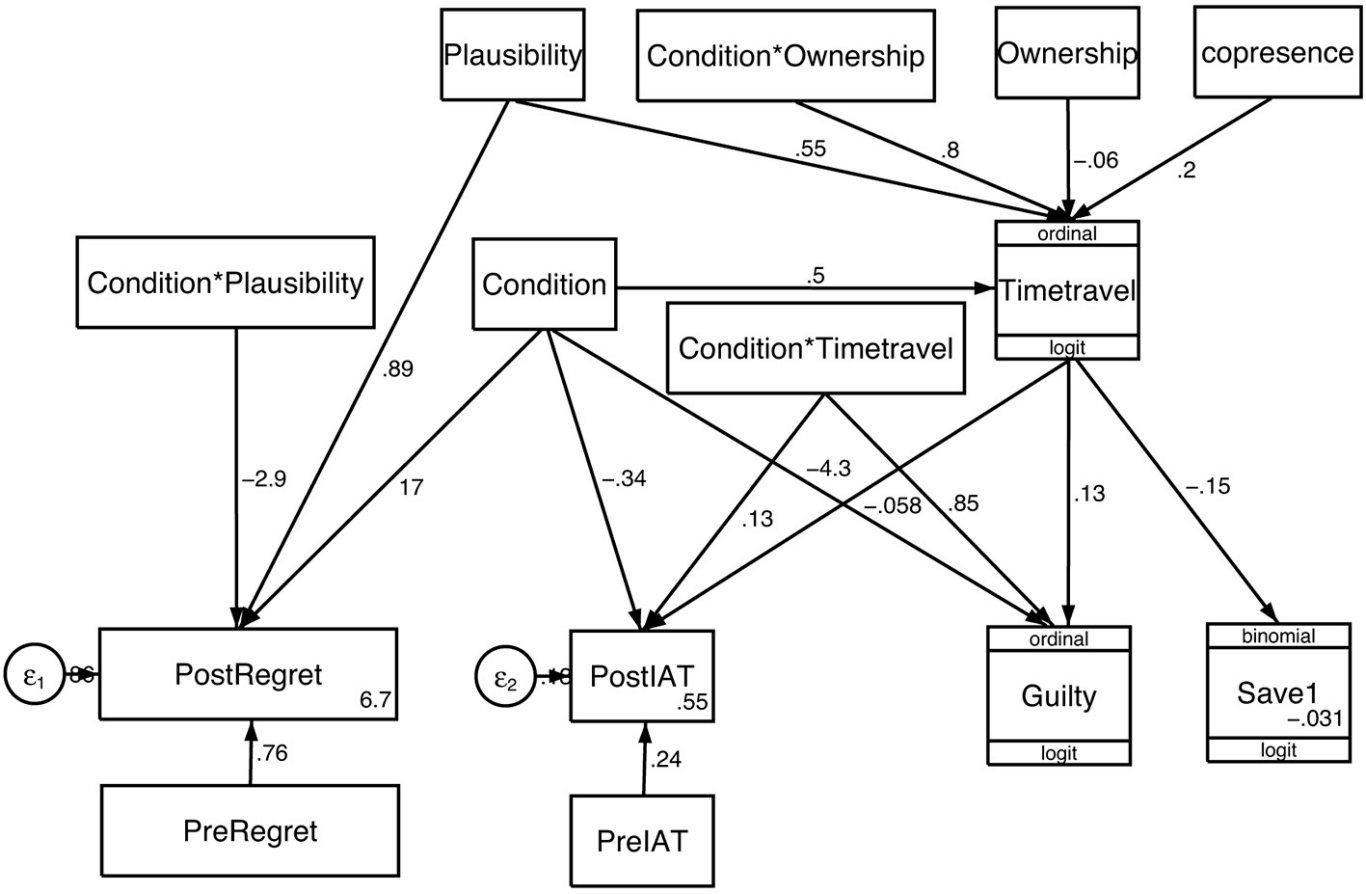
**Path analysis corresponding to Table 5**. The directional edges represent hypothesized directions of causality. The numbers on the edges are the coefficients of the linear predictor of the corresponding model fit. The variables in plain boxes are treated as linear normal models, and the specific model is otherwise shown in the remaining boxes.

Figure 3 shows the path diagram with statistics in Table 5. The path diagram was derived from the hypotheses (Sections “Hypotheses” and “Statistical Methods”). Paths that were not significant (for example, *placeillusion* to *timetravel*) have not been included. Where there is a significant interaction term (e.g., condition^*^timetravel) then following convention the main effects are included even if not significant. Table 5 gives the complete details about the path analysis. We consider each of the response variables in turn.

**Table 5:** **Path analysis corresponding to Figure 3, *n* = 32**.

	**Estimate of coefficient**	**Standard error**	***P***	**95% Confidence interval**	
***save1***					
*timetravel*	−0.151	0.065	0.021	−0.279	−0.023
*Constant*	−0.031	0.319	0.923	−0.656	0.594
***guilt***					
*timetravel*	0.131	0.284	0.644	−0.425	0.688
*Condition*	−4.302	1.777	0.015	−7.784	−0.820
Condition*timetravel	0.849	0.070	0.000	0.712	0.985
***postIAT***					
*timetravel*	−0.058	0.000	0.000	−0.059	−0.058
*Condition*	−0.344	0.250	0.169	−0.835	0.146
Condition*timetravel	0.130	0.018	0.000	0.095	0.165
*preIAT*	0.241	0.115	0.036	0.015	0.468
*Constant*	0.548	0.014	0.000	0.521	0.575
***timetravel***					
Condition*Ownership	0.804	0.259	0.002	0.297	1.311
*Ownership*	−0.060	0.050	0.230	−0.159	0.038
*plausibility*	0.548	0.071	0.000	0.408	0.687
*Condition*	0.503	0.754	0.504	−0.974	1.981
*copresence*	0.198	0.053	0.000	0.093	0.303
***PostRegret***					
*plausibility*	0.892	0.524	0.089	−0.136	1.920
*Condition*	16.832	3.486	0.000	9.999	23.665
*PreRegret*	0.764	0.119	0.000	0.530	0.997
Condition*plausibility	−2.901	1.226	0.018	−5.303	−0.499
*Constant*	6.679	12.222	0.585	−17.276	30.634

In the first column the dependent variables are shown in bold. Condition has Repetition = 0, Time Travel = 1. Constant refers to the intercept term of the linear predictor of each model equation. ^*^Refers to an interaction term. Standard Errors are robust and allow for non-independence (clustered on gender). P = 0.000 means P < 0.0005.

The variation in responses to the *timetravel* question is not explained by *Condition* alone. However, the path analysis shows that overall it is influenced by *plausibility, copresence* and the interaction between *Ownership* and *Condition*. The variable *timetravel* is positively associated with *plausibility, copresence* and in the Time Travel condition is positively associated with *Ownership*. However, there is no association with *Ownership* in the Repetition condition.

*Timetravel* is an endogenous variable in this model, and therefore can be entirely predicted within the model. The correlation between the fitted values of the linear predictor for *timetravel* and the observed values shows a good fit of the model to the data: Spearman's rho = 0.53 (*P* = 0.002).

Taking into account *PreRegret*, the *PostRegret* variable is positively influenced by the Time Travel condition, but the greater the *plausibility* the lower the value of *PostRegret* only in the Time Travel condition. In other words the greater illusion of reality the lower the rating of regret, provided that this was in the Time Travel Condition. The Spearman correlation of the fitted values of the linear predictor and the observed values is 0.60, *P* = 0.0003.

Taking into account the *PreIAT* score the *PostIAT* is negatively associated with the *timetravel* illusion in the Repetition condition, and positively in the Time Travel condition (i.e., there is a significant interaction effect between Condition and *timetravel*). In other words in the Time Travel condition greater levels of the illusion lead to an increase in implicit categorization of the self as moral. (Spearman's rho = 0.52, *P* = 0.002, for the correlation between fitted and observed values, as above).

**Figure 4:**
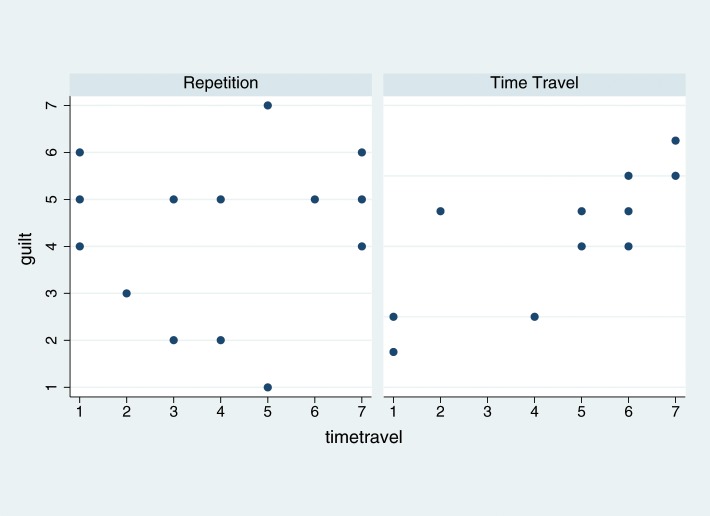
**Scatter diagram of *guilt* by *timetravel* for each of the two Conditions (Repetition and Time Travel)**.

The feeling of *guilt* is positively associated with the time travel illusion, but only in the Time Travel condition (Figures 3, 4). *Guilt* is an endogenous variable that can be predicted entirely within the model. The fitted values of the linear predictor for *guilt* are well correlated with the observed values (Spearman's rho = 0.62, *P* = 0.002).

**Figure 5:**
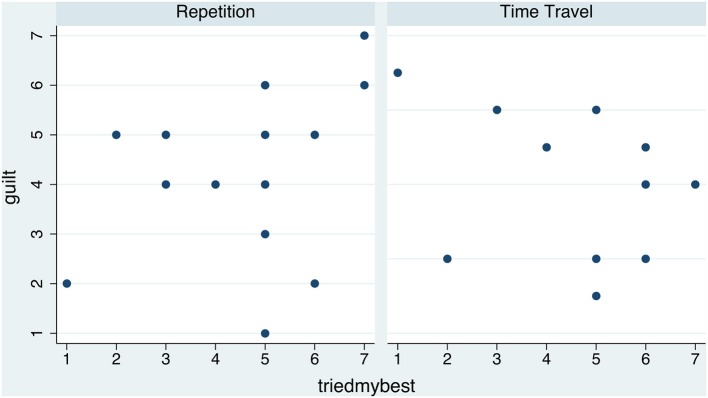
**Scatter diagram of *guilt* by *triedmybest* for each of the two Conditions (Repetition and Time Travel)**.

The results for *triedmybest* suggested that overall participants did try to save the visitors: this has a median score of 5 (IQR = 2). Moreover it is related to guilt. Figure 5 suggests that in the Time Travel condition the more that participants felt that they had tried their best the lower their guilt. An ordered logistic regression of *guilt* on Condition, *timetravel* and *triedmybest* allowing for interactions between these two variables and Condition supports this (Table 6). This shows that in the Repetition condition *triedmybest* is positively associated with *guilt*, but through the interaction between Condition and *triedmybest*, in the Time Travel condition the association is negative (coefficient = −1.1, *P* < 0.0005). Also in the Time Travel condition *timetravel* is positively associated with *guilt* (coefficient = 1.0, *P* < 0.0005). We show the coefficients since they are almost the same magnitude but opposite in sign. This means for example, that when in the Time Travel condition, participants have a strong subjective illusion of time travel, and a strong belief that they had tried their best then these two effects cancel out.

**Table 6:** **Ordered logistic regression for *guilt*, *n* = 32**.

	**Estimate of coefficient**	**Standard error**	***P***	**95% Confidence interval**	
***guilt***					
*Condition*	0.038	1.122	0.973	−2.162	2.238
*timetravel*	0.034	0.291	0.907	−0.536	0.604
*triedmybest*	0.542	0.027	0.000	0.490	0.595
Condition*timetravel	1.013	0.003	0.000	1.007	1.020
Condition*triedmybest	−1.065	0.045	0.000	−1.154	−0.976

Condition has Repetition = 0, Time Travel = 1. Constant refers to the intercept term of the linear predictor. ^*^Refers to an interaction term. Standard Errors are robust and allow for non-independence (clustered on gender). P = 0.000 means P < 0.0005.

The variable *save1* represents the propensity to save the 1 instead of the 5. This was negatively associated with *timetravel*. This variable is also endogenous and can be predicted from the model. The fitted values from the model correlate well with the observed values (Spearman's rho = 0.41, *P* = 0.02). Overall the stronger the illusion the more the tendency toward a utilitarian solution but also the greater the guilt.

## Discussion

The fundamental contribution of this paper has been to introduce a new method for the induction and exploration of the consequences of a time travel illusion using IVR. The application of the method has tackled a number of broad questions and the results point to some possible answers. However, our attempt to measure the “time travel” illusion was limited to a single question in the questionnaire, the reason being that since this is a new illusion it was not obvious which other questions to ask. A task of future work will be to improve the subjective measure, partially based on analysis of the interviews of participants after their experiences. However, notwithstanding this limitation the findings suggest a set of hypotheses for future work, each of which would ideally require a specific focused experimental study.

First, we have considered whether it is possible to induce such an illusion, and if so some of the consequences. Although the manipulation (Repetition or Time Travel) did not by itself influence the subjective illusion of time travel, it did so in conjunction with the illusion of body ownership, and plausibility, including the extent to which the virtual visitors were experienced as if they were real. Since this is an exploratory study we summarize this finding as a hypothesis: If in IVR with embodiment in a virtual body participants experience a sequence of events, and then are involved in those same events over again where they can also witness their past actions in the same virtual body that they previously had embodied, then they will experience this as time travel provided that they also have a strong sense of body ownership over that virtual body. Place illusion and plausibility will add to that illusion. This is premised on a strong sense of agency over the virtual body. Hence a future experiment would be designed to maximize the probability of high subjective body ownership, using multiple multisensory techniques—exploiting first person perspective, visuomotor synchrony, and visuotactile synchrony, discussed in Kokkinara and Slater (2014). Moreover there would also need to be an explicit “non-embodiment” condition as a control group with an attempt to minimize body ownership.

Our second hypothesis, and suggested future experimental study, is that the experience of such time travel may lead to participants implicitly accepting the notion that the past is mutable. This may lead to a re-evaluation of some of their own past actions that had unfortunate consequences and lessen the negative affect associated with these.

The moral IAT test had as moral categories: honesty, humility, altruism, modesty, sincerity, ethical; and for the immoral ones: deceptive, arrogant, cheater, egoism, vanity, corrupt. Our third hypothesis and suggested study is that the experience of the Time Travel condition together with a strong subjective sense of time travel would lead to a greater propensity to implicit self-classification as moral, with the meaning of this given by these categories. This result is compatible with Segovia et al. (2009) who carried out an experiment where people watched their self-representation avatars carry out immoral or moral actions. The results suggested that they became more immoral if they had seen their avatar carry out immoral actions compared to if they had seen their avatar carry out moral actions. From the results on *triedmybest* and its relationship to *guilt*, it is likely that most of the participants felt that they were behaving morally therefore resulting in this exploratory finding.

The fourth hypothesis and suggested study is that guilt feelings with respect to harm caused to others may be positively associated with the illusion of time travel in the Time Travel condition. We speculate that this may be because there is a greater likelihood of association of the time travel with actual history. We have seen that plausibility is positively associated with the illusion of time travel, but the causality may go both ways. If participants observe their own actions being replayed in the scenario, then since they remember having carried out those actions for sure, this may add to the illusion that these events actually happened. Therefore, the illusion that harm was done to people would be strengthened, and since that harm, whatever happened, was partly the responsibility of the actions (or non-actions) of the participant, there is greater room for guilt feelings. An experimental study would be required to test these ideas, and also to untangle the direction of causality.

The fifth hypothesis and study is that the illusion of time travel is associated with tending to agree with a utilitarian solution of the classic moral dilemmas. To explore this further, based on the current data, instead of using the variable *save1*, we replace that by the binary variable *footbridge*—which would be an extreme version of utilitarianism not normally chosen by the majority of respondents—i.e., deliberately and directly causing the death of 1 by pushing him into the path of the boxcar in order to save the 5. When this variable is used instead of *save1* then again there is a positive association with the time travel response (coefficient = 0.36, *P* = 0.001). Why people should become more utilitarian is not clear. It could be because the illusion of time travel leads to thoughts about future consequences in the sense that the deaths of five people may have a far greater impact on the future than the death of 1 (even though there may not be any absolute calculus that says that the death of x people is preferable to the death of y if x < y).

One very interesting finding is the behavior in the 1st time around. Recall that almost all the participants pressed the alarm button and that almost all the visitors were killed. This is in contrast to questionnaire studies, and also the two virtual reality studies that have reproduced moral dilemmas in IVR (Pan and Slater, 2011; Navarrete et al., 2012). In both, participants overwhelmingly saved the 5 (89 and 90% respectively in the relevant condition). It seems that giving participants any alternative action that seems to be related to solving the crisis, even though the action is useless (here pressing the alarm), is not helpful at all. In the classical and previous VR experiments typically one bystander would die out of the six, whereas here we have found it to be nearly all of them.

It is important to note that there were many other variables that were not statistically related to either the experimental conditions or to the illusion of time travel. For example, other than the differences in the number of actions, explainable by the situation of the different conditions, there seems to be no difference in actual behavior between the two conditions. It is also possible that the two IAT tests may have influenced one another. There were several variables with missing data that were not used in the current analysis, so we emphasize that our experiment was only exploratory, and its findings should be regarded as hypotheses for future work.

There is growing interest in the concept of “mental time travel,” that is the ability to project oneself to the past to relive past events in imagination (episodic memory), and to project also the future. There is evidence that episodic memory and the capacity to simulate future events share the same neural substrates, suggesting a common neurocognitive system (Botzung et al., 2008) and similar conclusions were drawn from a comprehensive linguistic analysis (Stocker, 2012). This has led to suggestions that episodic memory should be considered a part of a more general faculty of mental time travel, which includes key capabilities such as planning (Suddendorf and Corballis, 2007), or even suggestions for re-conceptualizing memory (Schacter et al., 2007). There is also a debate on whether this capability is unique to humans (Suddendorf and Busby, 2003; Suddendorf and Corballis, 2007).

However, if mental time travel is beneficial, in particular in the domain of self-improvement, then we suggest that our virtual time travel could also be beneficial, even though many of the research questions considered in the field of mental time travel are unrelated to our virtual time travel. At least since psychoanalysis, uncovering and to some extent reliving in imagination episodes from our personal history, have been thought to be beneficial. This has found its way into modern cognitive behavioral therapy, for example, in a method for the treatment of post-traumatic stress disorder (Ehlers et al., 2005). In this case an aspect of the treatment is to identify salient moments in the memory of the traumatic event, and then inventing an “alternative appraisal that the patient finds compelling” and actively incorporating the new appraisal into the trauma memory. This incorporation can be verbal, through imagery, through writing, or though acting out the memory.

Giving people the experience of time travel, and thereby an implicit learning that the past is mutable may be useful in releasing the grip of such past traumatic memories. Moreover, our approach opens the door to laboratory controlled experimental studies of the consequences of virtual time travel in this and other related domains.

### Conflict of interest statement

The authors declare that the research was conducted in the absence of any commercial or financial relationships that could be construed as a potential conflict of interest.

## Footnotes

^1^http://www.sonypictures.com/movies/looper/

^2^http://moral.wjh.harvard.edu

^3^http://www.nvisinc.com/product/products.html?reqview=spec&fid=a0QG0000009NGzCMAW&fname=nVisor%20SX111

^4^http://www.asus.com/Multimedia/HS1000W/

^5^http://www.stata.com

## References

[B1] Banakou D. Groten R. Slater M. (2013). Illusory ownership of a virtual child body causes overestimation of object sizes and implicit attitude changes. Proc. Natl. Acad. Sci. U.S.A. 110, 12846–12851 10.1073/pnas.1306779110 23858436 PMC3732927

[B2] Botzung A. Denkova E. Manning L. (2008). Experiencing past and future personal events: functional neuroimaging evidence on the neural bases of mental time travel. Brain Cogn. 66, 202–212 10.1016/j.bandc.2007.07.011 17881109

[B3] Cushman F. Young L. Hauser M. (2006). The role of conscious reasoning and intuition in moral judgment testing three principles of harm. Psychol. Sci. 17, 1082–1089 10.1111/j.1467-9280.2006.01834.x 17201791

[B4] Deutsch D. (2011). The Fabric of Reality. London: Penguin

[B5] Deutsch D. Lockwood M. (2009). The quantum physics of time travel, in Science Fiction and Philosophy–from Time Travel to Superintelligence, ed Scheider S. (Chichester; West Sussex: Blackwell Publishing Ltd), 322–334

[B6] Dowe P. (2000). The case for time travel. Philosophy 75, 441–451 10.1017/S0031819100000504

[B7] Ehlers A. Clark D. M. Hackmann A. McManus F. Fennell M. (2005). Cognitive therapy for post-traumatic stress disorder: development and evaluation. Behav. Res. Ther. 43, 413–431 10.1016/j.brat.2004.03.006 15701354

[B8] Greene J. D. Sommerville R. B. Nystrom L. E. Darley J. M. Cohen J. D. (2001). An fMRI investigation of emotional engagement in moral judgment. Science 293, 2105–2108 10.1126/science.1062872 11557895

[B9] Grey W. (1999). Troubles with time travel. Philosophy 74, 55–70 10.1017/S0031819199001047

[B10] Hauser M. Cushman F. Young L. Kang-Xing Jin R. Mikhail J. (2007). A dissociation between moral judgments and justifications. Mind Lang. 22, 1–21 10.1111/j.1468-0017.2006.00297.x

[B11] Kaplan D. (2009). Structural Equation Modeling: Foundations and Extensions. London: SAGE Publications Inc

[B12] Kilteni K. Normand J.-M. Sanchez Vives M. V. Slater M. (2012). Extending body space in immersive virtual reality: a very long arm illusion. PLoS ONE 7:e40867 10.1371/journal.pone.0040867 22829891 PMC3400672

[B13] Kokkinara E. Slater M. (2014). Measuring the effects through time of the influence of visuomotor and visuotactile synchronous stimulation on a virtual body ownership illusion. Perception 43, 43–58 10.1068/p7545 24689131

[B14] Llobera J. Sanchez-Vives M. V. Slater M. (2013). The relationship between virtual body ownership and temperature sensitivity. J. R. Soc. Interface 10, 1742–5662 10.1098/rsif.2013.0300 23720537 PMC4043162

[B15] Michotte A. (1963). The Perception of Causality. Oxford: Basic Books

[B16] Navarrete C. D. McDonald M. M. Mott M. L. Asher B. (2012). Virtual morality: emotion and action in a simulated three-dimensional trolley problem. Emotion 12, 364–370 10.1037/a0025561 22103331

[B17] Nemiroff R. J. Wilson T. (2013). Searching the Internet for evidence of time travelers. arXiv:1312.7128.

[B18] Pan X. Slater M. (2011). Confronting a moral dilemma in virtual reality: a pilot study. *BCS-HCI '11*, in Proceedings of the 25th BCS Conference on Human-Computer Interaction (Swinton), 46–51

[B19] Peck T. C. Seinfeld S. Aglioti S. M. Slater M. (2013). Putting yourself in the skin of a black avatar reduces implicit racial bias. Conscious. Cogn. 22, 779–787 10.1016/j.concog.2013.04.016 23727712

[B20] Perugini M. Leone L. (2009). Implicit self-concept and moral action. J. Res. Pers. 43, 747–754 10.1016/j.jrp.2009.03.015

[B21] Petkova V. I. Ehrsson H. H. (2008). If I were you: perceptual illusion of body swapping. PLoS ONE 3:e3832 10.1371/journal.pone.0003832 19050755 PMC2585011

[B22] Pomes A. Slater M. (2013). Drift and ownership towards a distant virtual body. Front. Hum. Neurosci. 7:908 10.3389/fnhum.2013.00908 24399960 PMC3872309

[B23] Sanchez-Vives M. V. Slater M. (2005). From presence to consciousness through virtual reality. Nat. Rev. Neurosci. 6, 332–339 10.1038/nrn1651 15803164

[B24] Schacter D. L. Addis D. R. Buckner R. L. (2007). Remembering the past to imagine the future: the prospective brain. Nat. Rev. Neurosci. 8, 657–661 10.1038/nrn2213 17700624

[B25] Segovia K. Y. Bailenson J. N. Monin B. (2009). Morality in tele-immersive environments, in IMMERSCOM '09 Proceedings of the 2nd International Conference on Immersive Telecommunications (Brussels), Article No. 17. 10.4108/ICST.IMMERSCOM2009.6574

[B26] Slater M. (2009). Place illusion and plausibility can lead to realistic behaviour in immersive virtual environments. Philos. Trans. R. Soc. Lond. 364, 3549–3557 10.1098/rstb.2009.0138 19884149 PMC2781884

[B27] Slater M. Spanlang B. Sanchez-Vives M. Blanke O. (2010). First person experience of body transfer in virtual reality. PLoS ONE 5:e10564 10.1371/journal.pone.0010564 20485681 PMC2868878

[B28] Steptoe W. Steed A. Slater M. (2013). Human tails: ownership and control of extended humanoid avatars. IEEE Trans. Vis. Comput. Graph. 19, 583–590 10.1109/TVCG.2013.32 23428442

[B29] Stocker K. (2012). The time machine in our mind. Cogn. Sci. 36, 385–420 10.1111/j.1551-6709.2011.01225.x 22268721

[B30] Suddendorf T. Busby J. (2003). Mental time travel in animals? Trends Cogn. Sci. 7, 391–396 10.1016/S1364-6613(03)00187-6 12963469

[B31] Suddendorf T. Corballis M. C. (2007). The evolution of foresight: what is mental time travel, and is it unique to humans? Behav. Brain Sci. 30, 299–313 10.1017/S0140525X07001975 17963565

[B32] Wright S. (1921). Correlation and causation. J. Agric. Res. 20, 557–585

[B33] Xu H. Bègue L. Bushman B. J. (2012). Too fatigued to care: ego depletion, guilt, and prosocial behavior. J. Exp. Soc. Psychol. 48, 1183–1186 10.1016/j.jesp.2012.03.00724882450

