# Supplementary material for: A method for generating an illusion of backwards time travel using immersive virtual reality—an exploratory study
Source: Front Psychol. 2014 Sep 2;5:943. doi: 10.3389/fpsyg.2014.00943 (PMC4151165; doi:10.3389/fpsyg.2014.00943)
Supplement: Supplementary file 4 [file DataSheet1.DOCX]

A method for generating an illusion of backwards time travel using immersive virtual reality - an exploratory study

Supplementary Material

Doron Friedman^1^, Rodrigo Pizarro^2^, Keren Or-Berkers^1^, Soléne Neyret^2^, Xueni Pan^3^, Mel Slater^2,4*^

^1^ Sammy Offer School of Communication, The Interdisciplinary Center, IDC Herzliya, Herzliya, Israel.

^2^ Event Lab, University of Barcelona, Barcelona, Spain.

^3^ Department of Computer Science, University College London, London, UK.

^4^ Institució Catalana de Recerca i Estudis Avançats (ICREA), Barcelona, Spain.

Supplementary Material

# 1. Background Data

There were 32 participants in each of the two experimental conditions (Repetition and Time Travel) 16 males and 16 females in each. The mean and standard errors of age are: Repetition: 22.2 ± 1.0 and Time Travel: 24.1 ± 1.5.

**Table S1** - Median and (Interquartile Range) of 4 background variables

| **Males** | **computer** | **programming** | **VR** | **gamesyear** | **gamesweek** |
| --- | --- | --- | --- | --- | --- |
| **Repetition** | **3.5** | **1** | **1** | **5** | **2** |
|  | (2.5) | (2) | (2) | (2.5) | (3) |
| **Time Travel** | **4** | **1.5** | **2.5** | **3** | **2** |
|  | (2) | (3.5) | (3) | (4.5) | (2) |
| **Females** |  |  |  |  |  |
| **Repetition** | **3.5** | **1** | **1.5** | **1** | **0** |
|  | (1) | (0.5) | (1) | (0) | (0) |
| **Time Travel** | **3.5** | **1** | **1.5** | **1** | **1** |
|  | (2.5) | (2.5) | (3) | (2.5) | (1.5) |

Data were collected on the following background variables, each rated on a 1-7 scale by the participants in their first visit to the laboratory:

- computer
  - the level of knowledge of computing.
- programming
  - the level of knowledge of computer programming
- vr
  - previous experience of VR
- games (over the year)
  - (1 = none, 2 = 1-5, 3 = 6-10, 4 = 11-15, 5 = 16-20, 6 = 21-25, 7 = >25)
- gamesweek (over the past week)
  - (1 = none, 2 = < 1, 3 = 1-3, 4 = 3-4, 5 = 5-7, 6 = 7-9, 7 = > 9)

Table S1 gives the medians and interquartile ranges, and it is clear that on the average there were no differences between the conditions or genders.

# 2. Procedures

In their first visit to the laboratory participants were given an information sheet about the experiment and asked to sign a consent form, and completed a demographic questionnaire (from which Table S1 was derived).

Next they were asked to complete two Implicit Association Tests (IAT) (Greenwald et al., 2003;Nosek et al., 2005), one to assess their view of their own morality (Perugini and Leone, 2009) and the other to test their general feelings of guilt (Xu et al., 2012). The tests are aimed to quantify the strength of associations between different concepts. The Moral IAT had 7 blocks (see (Nosek et al., 2007), Table 6.1, for the typical IAT procedure involving 7 blocks) with 24 trials per block and the categories were moral/immoral and me/others. The words for classification were honesty, humility, altruism, modesty, sincerity, ethical and correct for the moral category, and deceptive, arrogant, cheater, egoism, vanity, corrupt and dishonest for the immoral one. I, me, myself, mine, and theirs, others, they and them were used respectively for the me/others category.

The Guilty IAT had 7 blocks with 22 trials each. The categories were guilty/not guilty and me/others. The words to classify this IAT were innocent, irreproachable, blameless, clean, legal and legitimate for not guilty. Blameworthy, accusable, criminal, culpable, remorseful and guilty were used for guilty. The me/others category used the same words as the Moral IAT. The order of tests was balanced across participants. They were all told to sit in front of the screen and to try to perform the task as quickly as possible.

After the IAT tests the participants were asked to sit in a particular chair that was registered with one that they would appear to be sitting in during their first VR experience. They then donned the HMD and headphones. They were self-represented by a gender-matched virtual body, seen from first person perspective, and with the virtual body substituting their real body - in other words as they looked down towards their real body they would see the virtual body instead, and in the same seated posture. They were instructed to sit in a particular pose without moving the upper body, which matched the one represented by the virtual body. In this phase only the participant’s head was tracked.

After being positioned they heard all the pre-recorded instructions through the headphones. The instructions informed the participant that their task would start after the instructions, and that it consisted in observing the environment from the moment they heard an auditory cue ( a ‘beep’ sound) until they heard another such cue. They were told that everything they had done until the first cue was not part of the actual experiment, and were asked if they had any questions.

After this training phase and once any possible question had been answered, participants were immersed again in a virtual environment, this time in the virtual museum gallery. The gallery contained numerous paintings over two floors connected by an elevator. Six virtual humans entered the gallery, one every ten seconds. A number of visiting virtual human characters visited the ground floor and looked at the paintings except for the second visitor, who took the elevator and went to the upper level. All the virtual characters followed a predetermined path along the gallery. After 450 seconds, an auditory cue was played and the screen faded to black. The participants were then instructed to report their estimation of elapsed time regarding between the two cues.

Next the HMD was removed from the participant’s head and they were asked to think about the three decisions they regretted most in their lives. They were then provided with a pen and a folded paper sheet, and were told that the experimenters would leave the room for a few minutes. During that absence they were to write a short description of the decisions they regretted the most in their lives on the inside part of the paper sheet and a score from 1-100 on the outer part, where 1 meant they did not regret this past decision at all and 100 meant that they regretted it to the maximum. They were assured that nobody would ever read the descriptions and that they would be stored inside an envelope in a safe place, opened only in the second phase of the study.

Next they were asked to read an explanation about time travel and then to summarise this. The explanation of time travel emphasised that time travellers to the past can change history as if the original history had never happened.

The second phase of the study was approximately one week later for each participant. They first completed the written consent form. Next, they completed the Autonomic Perceptions Questionnaire (Mandler et al., 1958;Shields, 1984) - a visual-analogue scale of various physiological indicators where high scores indicate greater subjective awareness of somatic state, found to be positively correlated with anxiety, heart rate, skin conductance responses, respiration, face temperature, and blood volume.

Participants then donned the tracking suit and the tactile vibrators, the head-mounted display and the headphones, and they remained in a standing position. The gallery virtual environment was displayed, and pre-recorded instructions were given through the headphones. The gallery was the same as the one seen in the first phase except that the virtual visitors behaved differently and participants were standing in front of a futuristic looking workbench with several control buttons. This control workbench had two buttons marked with up and down arrows and there was a third button in red. The participants were embodied in a gender-matched virtual body seen from their own first person perspective that substituted their real body and that moved in synchrony with their movements. They could also see this body in a mirror mounted as part of the workbench.

As part of an acclimatisation process participants were asked to look around the gallery and at themselves in the mirror and to describe what they saw. They were then informed that their task was to take visitors to the upper floor if they requested this, to watch over them and to keep track of how many people were on each floor. They were taught how to operate the buttons to control the elevator in order to make it go up and down, and shown how to use the red alarm button that was also on the workbench. They were shown that the alarm button would freeze the lift and also emit an alarm sound with a red flashing light, and also that it was a toggle - so that if the alarm was on, pressing the button would turn it off, and vice versa. They were told that if the alarm went off for no apparent reason they should call out “False Alarm” and press the alarm button to stop it. Recall that when pressing any button they would feel a vibrotactile sensation on the hand that was used.

After these instructions, the first trial, common to all conditions started. Six virtual visitors entered the virtual gallery at a rate of one every twelve seconds. Each of them asked the participant to take them to the upper floor except for the second visitor, who asked what the time was, and stayed on the ground floor. Finally, a seventh visitor looking and acting the same as the previous ones entered the gallery and asked the participant to take him to the upper floor. Once the elevator reached the upper floor, this visitor started shooting all the visitors that were there, one shot every two seconds.

The shooting could stop under two conditions: if either all the visitors had been shot or if the elevator was sent down. If the elevator reached the ground floor and there were other visitors there, the shooting could start again.

The experimenters always waited for seven seconds after the end of the shooting and then activated the end of the trial to start the next one, with a total of three trials per participant.

Immediately after the end of the three trials the HMD, the tactile vibrators and the headsets were removed from the participant’s head and they were asked to perform the APQ test again. Next they completed the two IATs in the reverse order to that of the first phase of the experiment a week earlier. Finally, they completed a questionnaire concerned with body ownership and various aspects of the experience, the most important of which was whether they had the sensation of travelling back in time.

After completion of the questionnaires the tracking suit was removed. Then they were asked to remember the decisions they wrote in the first phase of the study (i.e., the decisions they regretted the most in their lives). They were able to open the envelope from the previous week so that they could read the descriptions they had previously written, but not the scores. They were requested to score those decisions again on the same 1-100 scale, and then to compare their new scores with the original ones. At that point the experimenters turned the video camera on and asked the participant how he or she felt about the decisions and if he or she felt that the consequences could still be changed. If there was a large change in the scores, participants were asked their opinion of the reason.

Afterwards two videos were shown to the participants in a random order. Each video showed an avatar saying an untrue sentence, invented by the experimenters: avatar 1 “The minimum salary in Spain rose 5.23% in the 60s”; avatar 2 “The minimum salary in Spain rose 3.57% in the 50s”. In one of the videos the avatar was the same one they embodied during the IVE phase and the other avatar was only similar in gender and ethnic group. Participants were instructed to watch the videos and select the avatar they trusted the most.

Finally, participants were interviewed.

# 3. Questionnaires

## Perception Questions

In what follows the term 'virtual body' refers to the body that you saw when you looked towards yourself which you also saw reflected in the mirror in front of you. Indicate your level of agreement with each statement below, on the scale from 1 to 7. (1=Strongly disagree, 7=Strongly disagree unless otherwise specified)

Even though the virtual body I saw did not look like me, I had the sensation that the virtual body I saw in the mirror was mine.

Even though the virtual body I saw did not look like me, I had the sensation that the virtual body, that I saw when I looked down at myself, was mine.

(1=Never, 7=Almost all the time)

I felt that the virtual body that I saw was someone else.

(1=Never, 7=Almost all the time)

Overall even though the virtual body I saw did not look like me I had the sensation that the virtual body I saw was my body.

The virtual body moved according to my movements.

I had the sensation of being in the gallery.

There were times when the gallery was more real for me than the laboratory in which everything was really taking place.

##

## The Visitors

The following questions concern the visitors to the gallery.

How much did you find yourself responding to the visitors as if they were real people?

(1=Not at all, 7=Very much)

How much responsibility did you feel for the safety of the visitors once the gunman started shooting? (1=None, 7=Very much)

I tried my best to save the visitors from the shooting. (1=Not at all, 7=Very much)

## The Event

These questions are about your feelings RIGHT NOW.

Which best describes your feeling right now after this experience?

 None

 Happy

 Sad

 Relief

 Surprise

 Disgust

 Fear

 Anxiety

 Angry

 Other: 

Overall how many times did you push the alarm button? (Please answer with a number)

Overall how many shots were fired? (Please answer with a number).

I felt I relived the same situation several times. (1=Not at all, 7=Very much)

The overall experience was more like...

(1=Replaying a video game, 7=Experiencing time travel)

How satisfied are you with the final outcome?

(1=Not satisfied at all, 7=Very satisfied)

Please explain

(Open answer)

Do you feel any guilt about what happened to the visitors?

(1=Not at all, 7=Very much)

To what extent did you feel you were only solving a puzzle?

(1=Not at all, 7=All the time)

##

## Questions about Some Short Stories

You will now read five similar (but not the same) short stories. Please answer the question after each story. In this section, all possible answers were yes or no options, except for the last question, which was an open answer.

## Scenario 1

You are the operator of a lift in a gallery that shows paintings. You can see the gallery and the lift directly and you operate the lift using two buttons, the up and the down button. You are not in the lift but operating it from a small distance away. The lift itself is a platform surrounded by glass walls. There are two floors in the gallery (ground floor and upper floor) and the only access to the upper floor is by the lift. A person standing on the lift after it has reached the upper floor cannot see the area of the ground floor where visitors might be looking at the paintings. There are 5 visitors on the upper floor and 1 on the ground floor. One other person steps on the lift and you push the switch to take this person to the upper floor. When the lift arrives at the upper floor, this person takes out a gun and starts shooting at the 5 people on the upper floor. One of the people on the upper floor is immediately killed, and the remaining 4 are clearly in danger of their lives. The attacker is still on the lift. If you move the lift downstairs immediately you will save the 4 people on the upper floor. However by doing so you are putting the life of the one person on the ground floor in danger.

Would you push the down button?

## Scenario 2

An empty boxcar is running out of control down a track. In its path are five people standing on the track; these people are not aware of the oncoming danger. If the boxcar continues, it will kill all five people. You are standing next to a switch. If you flip the switch, it will cause the boxcar to turn off of the main track and onto a side track. On the side track there is one person who is also unaware of the boxcar. If the boxcar goes down this side track, the one person will die but the five people on the main track will survive.

Would you flip the switch?

## Scenario 3

An empty boxcar is hurtling out of control down a track towards five people. If the boxcar continues, it will kill all five people. You are on a bridge over the tracks. The boxcar will pass under the bridge before it reaches the five people. You can stop the boxcar by dropping a heavy weight in front of it. Standing next to you is a man wearing a heavy backpack. If you push him over the bridge, he will land in front of the boxcar and stop it before it reaches the five people ahead. This man will, however, die.

Would you push the man over the bridge?

## Scenario 4

You are the operator of a lift in a gallery that shows paintings. You can see the gallery and the lift directly and you operate the lift using two buttons, the up and the down button. You are not in the lift but operating it from a small distance away. The lift itself is a platform surrounded by glass walls. There are two floors in the gallery (ground floor and upper floor) and the only access to the upper floor is by the lift. A person standing on the lift after it has reached the upper floor cannot see the area of the ground floor where visitors might be looking at the paintings. There is 1 visitor on the upper floor and 5 on the ground floor. One other person steps on the lift and you push the button to take this person to the upper floor. When the lift arrives at the upper floor, this person takes out a gun and starts shooting at the person on the upper floor. The person on the upper floor is immediately injured, and is clearly in danger of his life. The attacker is still on the lift. If you move the lift downstairs immediately you will save the person on the upper floor. However by doing so you are putting the life of the 5 people on the ground floor in danger.

Would you push the down button?

## Scenario 5

An empty boxcar is running out of control down a track. In its path is one person standing on the track; this person is not aware of the oncoming danger. If the boxcar continues, it will kill this person. You are standing next to a switch. If you flip the switch, it will cause the boxcar to turn off of the main track and onto a side track. On the side track there are five people who are also unaware of the boxcar. If the boxcar goes down this side track, the five people will die but the one person on the main track will survive.

Would you flip the switch?

## Final comments

Have you seen any version of this experiment before? (e.g., on BBC Horizon)

Overall please describe how you are feeling now, taking into account your whole experience, and why you might be feeling that way. Your comments will be very helpful for us to understand the results.

# 4. Further information on the IATs

**4.1 Moral IAT**

During the first IAT block, the participant was asked to categorize visual stimuli into the two target categories, namely “Me” and “Others” (Table S2). The stimuli were words appearing in the middle of the screen for the participant to sort into the appropriate category. In the second block, the participant was trained to press one button for “Moral” attributes and the other button for “Immoral” attributes. The stimuli (Table S3) were also words appearing in the centre of the screen. The third and fourth blocks combined the target and the attribute discrimination that were subdivided into two blocks of 24 trials each. The subsequent fifth block reversed the target discrimination and the sixth and seventh blocks combined again the attribute and the previously reversed target discrimination.

The IAT followed the standard IAT 7 blocks procedure (Tables S2-S4). Participants were instructed to “respond rapidly, while occasional errors are acceptable”; categorization errors were identified with a red “X” below the stimulus item and participants had to correct the response before continuing to the next trial. All category labels, which assigned to the right or left response key, were displayed in the right or left upper screen corner throughout all tasks. To emphasize the distinction of the labels and stimuli of the target concept, “Moral/Immoral” labels appeared in light blue font, and “Me/Others” labels and items in yellow font, all on a black background; instructions were presented in white. The target and attribute stimuli were alternated in the combined blocks.

Stimuli for “Moral/Immoral” category were similar to those used in previous studies (Perugini et al. 2009)

**Table S2** Blocks structure of the Moral IAT

| **7 blocks structure** |  |
| --- | --- |
| first block: me/others | 10 trials (5 stimuli for "me", 5 for "others", each stimulus is presented once) |
| second block: moral/immoral | 14 trials (7 stimuli for "moral", 7 for "immoral", each stimulus in presented once) |
| third block (combined, me+moral, others+immoral) | 24 trials (each stimuli is presented once) |
| fourth block (combined, me+moral, others+immoral) | 24 trials (each stimuli is presented once) |
| fifth block (reverse buttons for me/others) | 10 trials (5 stimuli for "me", 5 for "others", each stimulus is presented once) |
| sixth block (combined, me+immoral, others+moral) | 24 trials (each stimuli is presented once) |
| seventh block (combined, me+immoral, others+moral) | 24 trials (each stimuli is presented once) |

**Table S3** Words stimuli for Me/Others category in Moral IAT

| **Me** | **Others** |
| --- | --- |
| Me | You |
| Mine | Theirs |
| With me | Others |
| My | They |
| To me | Those |

**Table S4** Words stimuli for Moral/Immoral category in Moral IAT

| **Moral** | **Immoral** |
| --- | --- |
| Honesty | Lie |
| Loyalty | Arrogance |
| Generosity | Cheater |
| Altruism | Egoism |
| Sincerity | Vanity |
| Modesty | Corrupt |
| Correct | Dishonest |

**4.2 Guilt IAT**

The guilt IAT was very similar to the moral one. The major change was the stimuli, which used words related to feelings of guilt (Tables S5-S7).

**Table S5** Blocks structure of the Guilt IAT

| **7 blocks structure** |  |
| --- | --- |
| first block: me/others | 10 trials (5 stimuli for "me", 5 for "others", each stimulus is presented once) |
| second block: not guilty/guilty | 12 trials (6 stimuli for "not guilty", 6 for "guilty", each stimulus in presented once) |
| third block (combined, me+not guilty, others+guilty) | 22 trials (each stimuli is presented once) |
| fourth block (combined, me+not guilty, others+guilty) | 22 trials (each stimuli is presented once) |
| fifth block (reverse buttons for me/others) | 10 trials (5 stimuli for "me", 5 for "others", each stimulus is presented once) |
| sixth block (combined, me+guilty, others+not guilty) | 22 trials (each stimuli is presented once) |
| seventh block (combined, me+guilty, others+not guilty) | 22 trials (each stimuli is presented once) |

**Table S6** Words stimuli for Me/Others category in Guilt IAT

| **Me** | **Others** |
| --- | --- |
| Me | You |
| Mine | Theirs |
| With me | Others |
| My | They |
| To me | Those |

**Table S7** Words stimuli for Moral/Immoral category in Guilt IAT

| **Not Guilty** | **Guilty** |
| --- | --- |
| Innocent | Reprehensible |
| Irreprochable | Acusable |
| Unimpeachable | Criminal |
| Clean | Culpable |
| Legal | Offender |
| Legitimate | Shameful |

# 5. Time travel priming

The following explanation was given to all participants during their first visit, irrespective of condition.

Have you ever thought about time travel, the ability to go back to the past? What would be the consequences if this were possible? Perhaps you would like to think of some event in which you were involved where the outcome was not what you wanted. Now you think to yourself “If only I had done …” something different I could have changed how things turned out.

Time travel makes this possible. Did you know that if you were able to go back and change the past then what happened before never actually happened? If you went back to the past you could even visit a place and time where you had been before and perceive yourself carrying out actions and doing whatever you did then. You might be able to even change the consequences of your past actions by interfering with events in the past.

There are different theories of time travel. In some theories your past self would see your time travel self, and you could interact with your past self. In other theories your time travel self would not be visible to your earlier self.

But the most important thing of all is that if you travelled back in time and changed something, then that change could have repercussions and change the future.

There is a famous science fiction story where a time traveller went so far back in time that there were still dinosaurs roaming the earth. By accident he stepped on a tiny creature and killed it. When the time traveller returned to the present, there were no humans – only seemingly lizard-like creatures. When he went back to the past he had inadvertently killed a precursor of mammals, and so human beings never developed.

Change the past and you change the future. What happened, now never happened.

When you change the past what happened before never happened. History itself has changed.

Do you think that you will ever experience time travel?

# 6. The interview

After completing all the questionnaires participants were interviewed. The following questions were asked, and the results will be analyzed for a subsequent report.

Tell us in your own words what happened.

Tell us about your feelings now.

Did you have the feeling of going back in time?

Could you see at any point avatars standing next to you? ^[[1]](#footnote-1)^

How would you describe the avatar that was standing next to you?2

What made you recognize the avatar as your previous self?^[[2]](#footnote-2)^

What did you feel when the visitors in the top floor got shot?

What did you feel when the visitors in the ground floor got shot?

Were you afraid you could get shot?

Were you afraid one of your previous selves could get shot? 2

To what extent did you feel scared when the visitors in the top floor were shot?

To what extent did you feel scared when you were shot? (if participant got shot)

Please comment anything you want to say about the experience.

# References

Greenwald, A.G., Nosek, B.A., and Banaji, M.R. (2003). Understanding and using the Implicit Association Test: I. An improved scoring algorithm. *Journal of Personality and Social Psychology* 85**,** 197.

Mandler, G., Mandler, J.-M., and Uviller, E.-T. (1958). Autonomic feedback: The perception of autonomic activity. *Journal of Abnormal and Social Psychology* 56**,** 367-373.

Nosek, B.A., Greenwald, A.G., and Banaji, M.R. (2005). Understanding and using the Implicit Association Test: II. Method variables and construct validity. *Personality and Social Psychology Bulletin* 31**,** 166-180.

Nosek, B.A., Greenwald, A.G., and Banaji, M.R. (2007). The Implicit Association Test at age 7: A methodological and conceptual review. *Automatic processes in social thinking and behavior***,** 265-292.

Perugini, M., and Leone, L. (2009). Implicit self-concept and moral action. *Journal of Research in Personality* 43**,** 747-754.

Shields, S.A. (1984). Reports of bodily change in anxiety, sadness, and anger. *Motivation and Emotion* 8**,** 1-21.

Xu, H., Bègue, L., and Bushman, B.J. (2012). Too fatigued to care: ego depletion, guilt, and prosocial behavior. *Journal of Experimental Social Psychology* 48**,** 1183-1186.

1. Only asked in time travel condition [↑](#footnote-ref-1)
2. The actual question was formulated based on the words chosen by the participant [↑](#footnote-ref-2)
